# Construction of an infectious clone for enterovirus A89 and mutagenesis analysis of viral infection and cell binding

**DOI:** 10.1128/spectrum.03332-23

**Published:** 2024-03-05

**Authors:** Jingjing Yan, Min Wang, Xiaohong Li, Jun Fan, Rui Yu, Miaomiao Kang, Yong Zhang, Jianqing Xu, Xiaoyan Zhang, Shuye Zhang

**Affiliations:** 1 Shanghai Public Health Clinical Center, Fudan University, Shanghai, China; 2 WHO WPRO Regional Polio Reference Laboratory, National Health Commission Key Laboratory for Biosafety, National Institute for Viral Disease Control and Prevention, Chinese Center for Disease Control and Prevention, Beijing, China; 3 Clinical Center for Biotherapy, Zhongshan Hospital, Fudan University, Shanghai, China; Thomas Jefferson University, Philadelphia, Pennsylvania, USA

**Keywords:** enterovirus A89, reverse genetics, infectious clone, subgenomic replicon, site mutations

## Abstract

Enterovirus A89 (EV-A89) is an unconventional strain belonging to the *Enterovirus A* species. Limited research has been conducted on EV-A89, leaving its biological and pathogenic properties unclear. Developing reverse genetic tools for EV-A89 would help to unravel its infection mechanisms and aid in the development of vaccines and anti-viral drugs. In this study, an infectious clone for EV-A89 was successfully constructed and recombinant enterovirus A89 (rEV-A89) was generated. The rEV-A89 exhibited similar characteristics such as growth curve, plaque morphology, and dsRNA expression with parental strain. Four amino acid substitutions were identified in the EV-A89 capsid, which were found to enhance viral infection. Mechanistic studies revealed that these substitutions increased the virus’s cell-binding ability. Establishing reverse genetic tools for EV-A89 will significantly contribute to understanding viral infection and developing anti-viral strategies.

**IMPORTANCE:**

*Enterovirus A* species contain many human pathogens and have been classified into conventional cluster and unconventional cluster. Most of the research focuses on various conventional members, while understanding of the life cycle and infection characteristics of unconventional viruses is still very limited. In our study, we constructed the infectious cDNA clone and single-round infectious particles for the unconventional EV-A89, allowing us to investigate the biological properties of recombinant viruses. Moreover, we identified key amino acids residues that facilitate EV-A89 infection and elucidate their roles in enhancing viral binding to host cells. The establishment of the reverse genetics system will greatly facilitate future study on the life cycle of EV-A89 and contribute to the development of prophylactic vaccines and anti-viral drugs.

## INTRODUCTION

Human enteroviruses (EVs) are members of the *Enterovirus* genus in the Picornaviridae family. EVs are non-enveloped, single-stranded RNA viruses with a positive-sense genome of approximately 7,500 nucleotides (1). The EV genome consists of an open reading frame (ORF) flanked by 5′ and 3′ untranslated regions (UTRs). The ORF encodes a single large polyprotein that is cleaved by viral proteases into four viral capsid proteins (VP1–VP4) and seven non-structural proteins (2A–2C and 3A–3D). The 5′-UTR contains an internal ribosome entry site essential for translation initiation. The 3′-UTR forms conserved secondary and tertiary structures involved in RNA replication. EV infections are usually self-limiting, but they can also cause outbreaks of various illnesses such as acute flaccid paralysis (AFP), acute hemorrhagic conjunctivitis, aseptic meningitis, encephalitis, myocarditis, and hand, foot, and mouth disease (HFMD) (2–4). Thus, EV infection-associated diseases pose a great threat to public health, particularly in young children.

Human EVs have been classified into four species, including *EV-A*, *EV-B*, *EV-C*, and *EV-D* (5, 6). Over 100 serotypes of human EVs have be identified. Among them, *EV-A* consists of at least 25 serotypes and is further classified into conventional cluster and unconventional cluster (7, 8). Conventional cluster serotypes, such as enterovirus A71 (EV-A71), coxsackievirus A16 (CV-A16), coxsackievirus A6 (CV-A6), and coxsackievirus 10 (CV-A10) are the major causative agents of HFMD. In contrast, enterovirus A76 (EV-A76), enterovirus A89 (EV-A89), enterovirus A90 (EV-A90), and enterovirus 91 (EV-A91), previously isolated from AFP patients, form a distinct phylogenetic clade within *EV-A* and are referred to as the unconventional cluster. Prototype EV-A89 was initially isolated from stool specimens of an AFP patient in Bangladesh in 2000 (7). Several other EV-A89 strains have been isolated from AFP patients, acute gastroenteritis patients, or healthy individuals during disease surveillance activities in Bangladesh (7, 9), India (10–13), and Egypt (14). In China, the first EV-A89 strain (KSYPH-TRMH22F/XJ/CHN/2011, GenBank number KT277550.1, also as the reference strain (Ref) for this study) was isolated in 2011 from a contact of an AFP patient during AFP case surveillance in the Xinjiang Uygur Autonomous region (15). However, due to the limited study, the biological and pathogenic properties of EV-A89 and other unconventional *EV-A* viruses remain unclear. Therefore, further studies are necessary to elucidate the mechanisms of infection and to develop prophylactic vaccines and anti-viral drugs.

Reverse genetics is a convenient method for studying the pathogenesis and virulence of viruses. The infectious clones have been established for many EV serotypes, including poliovirus (16), EV-A71 (17–19), CV-A6 (20), CV-A10 (21, 22), CV-A16 (23), CV-B3 (24), CV-B5 (25), and EV-D68 (26). These clones have been used to validate the biological characteristics of the rescued viruses. However, to date, no infectious clone has been reported for EV-A89 or any other serotypes belonging to the unconventional *EV-A* cluster. In addition, the single-round infectious particles (SRIPs) for enteroviruses are valuable for studying viral pathogenesis (27–29). In this study, we have established the first infectious clone and SRIPs for EV-A89 and revealed the effects of capsid mutations on viral activities.

## RESULTS

### Recovery and characterization of recombinant EV-A89 derived from infectious clone

**Fig 1:**
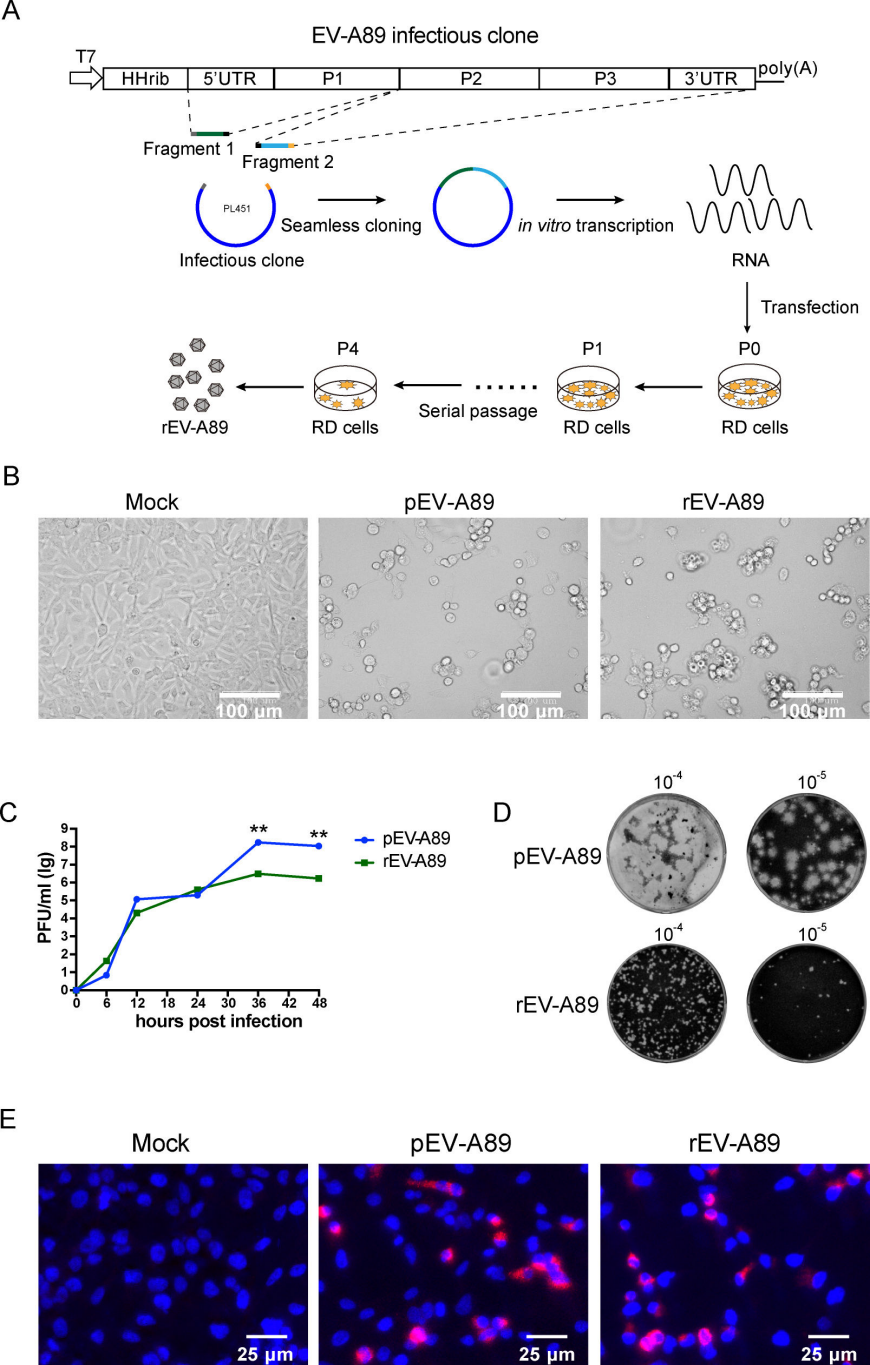
Recovery and characterization of recombinant EV-A89 derived from the full-length infectious clone. (**A**) The carton depicted the structure of the EV-A89 full-length infectious clone and the strategy to rescue recombinant EV-A89. The EV-A89 infectious clone was established by seamless cloning. After *in vitro* transfection, the genomic RNA was transfected into RD cells. By serial passage, recombinant EV-A89 was rescued successfully. (**B**) Cytopathic effects in RD cells infected with parental EV-A89 (pEV-A89) and recombinant EV-A89 (rEV-A89) at an MOI of 1 for 12 hpi. Scale bar, 100 µm. (**C**) Growth curves of the pEV-A89 and rEV-A89. (**D**) Plaque morphology of pEV-A89 and rEV-A89. Monolayers of RD cells in six-well plates were infected with pEV-A89 and rEV-A89. The cell monolayers were overlaid with 1.2% Avicel and stained with crystal violet at 72 hpi. (**E**) Immunofluorescence assay analysis of the dsRNA expression in RD cells. RD cells were infected with pEV-A89 and rEV-A89 at an MOI of 1. At 7 hpi, cells were fixed and stained with anti-dsRNA J2 antibody, followed by Alexa Fluor 594 conjugated goat anti-mouse IgG (H + L). 4',6-diamidino-2-phenylindole (DAPI) was used to visualize the nuclei (red, dsRNA; blue, nuclei). Scale bar, 25 µm. The significance levels are indicated by **P < 0.01.

The overall strategy to construct the infectious clone of EV-A89 was shown in Fig. 1A. The EV-A89 viral RNA was extracted from the viral supernatants and subjected to reverse transcription using oligo (dT) primers. Two overlapped fragments covering the whole genome were amplified and seamlessly cloned into the PL451 vector to construct the EV-A89 infectious clone. The sequences of the infectious clone were identified by Sanger sequencing. To rescue the recombinant enterovirus A89 (rEV-A89), the full-length infectious clone of EV-A89 was transcribed into RNA using T7 polymerase *in vitro*, and then the RNA was transfected into rhabdomyosarcoma (RD) cells. The supernatants of the transfected cells at 48 h post-transfection were collected as the passage 0 (P0) viruses. Serial passages of the P0 virus were performed by inoculating fresh RD cells to rescue live rEV-A89 (Fig. 1A). Comparative analysis was conducted between parental enterovirus A89 (pEV-A89) and rEV-A89 regarding cytopathic effects (CPEs) on RD cells. Both viruses induced typical CPE (Fig. 1B). Then the growth kinetics of the pEV-A89 and rEV-A89 were evaluated by infecting RD cells with each virus at a multiplicity of infection (MOI) of 0.01. Supernatants were harvested at various time points (0, 6, 12, 24, 36, and 48 hpi). The results showed that rEV-A89 exhibited similar growth kinetics to pEV-A89 before 24 hpi. However, its replication level was lower than pEV-A89 at 36 and 48 hpi (Fig. 1C). Plaque morphology was further examined, revealing that rEV-A89 produced smaller plaques compared to pEV-A89 (Fig. 1D). We speculate that the quasi-species present in pEV-A89 may possess enhanced adaptability in cell culture conditions, which may contribute to the differences in replication levels seen between rEV-A89 and pEV-A89. Immunofluorescence assay was performed to detect the expression of dsRNA in RD cells infected by pEV-A89 and rEV-A89. As shown in Fig. 1E, positive intracellular expression of dsRNA was observed in both pEV-A89- and rEV-A89-infected cells, indicating comparable levels of dsRNA expression. These results indicated that rEV-A89 displayed similar CPE and dsRNA expression to pEV-A89 but exhibited a slightly lower replication level.

### The mutant sites on EV-A89 capsid were indispensable for viral infection

**Fig 2:**
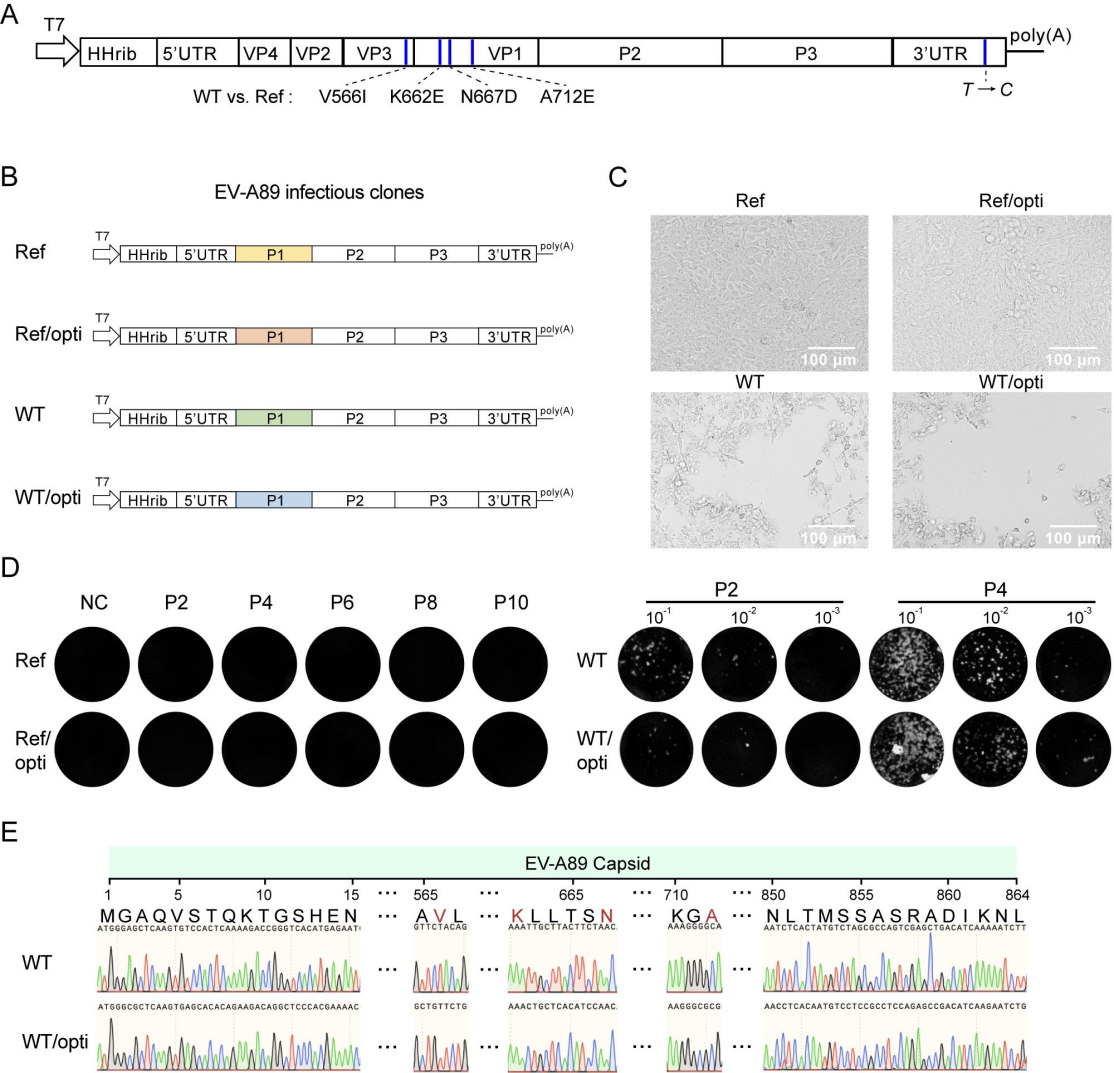
The mutant sites on EV-A89 capsid facilitated viral infection. (**A**) The carton described the mutant sites in the EV-A89 genome. There were four amino acid substitutions in the capsid region, including one in VP3 (V566I) and three in VP1 (K662E, N667D, and A712E). At the 3′-UTR region, one nucleotide mutation (*T* to *C*) was observed. (**B**) Construction of full-length infectious clones for EV-A89 Ref, EV-A89 Ref with P1 codon optimization (Ref/opti), and EV-A89 WT with P1 codon optimization (WT/opti). The infectious clone for EV-A89 WT was used as a control. (**C**) Cytopathic effects on RD cells inoculated with serial passaged EV-A89 derived from the four infectious clones. Scale bar, 100 µM. (**D**) Plaque assay for serial passaged EV-A89 derived from the four infectious clones. RD cells were inoculated with the serial passaged EV-A89 viruses derived from Ref and Ref/opti infectious clones (P2, P4, P6, P8, and P10) and the 10-fold serial diluted P2 and P4 viruses derived from WT and WT/opti infectious clones. One hour later, RD cells were overlaid with 1.2% Avicel and stained with crystal violet at 72 hpi. (**E**) Sanger sequencing of the capsid sequences of EV-A89 rescued from WT and WT/opti infectious clones.

In our previous attempt, we synthesized the whole genome of the EV-A89 reference strain (GenBank number KT277550.1) according to the published sequences from National Center for Biotechnology Information (NCBI) (15). The synthesized genome was cloned into the PL451 vector, and after *in vitro* transcription, the genome RNA was introduced into RD cells to rescue viruses. However, we failed to acquire the recombinant EV-A89 using this method. Here, we constructed an infectious cDNA clone from the wild-type (WT) EV-A89 and successfully rescued rEV-A89. Consequently, we hypothesized that there might be differences in the sequence of the EV-A89 genome between the WT and Ref strains. Indeed, upon aligning the entire genomes of WT and Ref, we identified four amino acid substitutions, VP3-V242I (V566I), VP1-K94E (K662E), VP1-N99D (N667D), and VP1-A144E (A712E), in the capsid region and one nucleotide mutation (*T* to *C*) in 3′-UTR (Fig. S1; Fig. 2A). To investigate the significance of these four amino acid mutations for viral infection, we created a chimeric infectious clone by replacing the capsid region of EV-A89 WT with that of EV-A89 Ref (Fig. 2B). Additionally, since we previously found that codon optimization improved EV-A89 capsid expression (8, 30) (Fig. S2), we also created infectious clones of EV-A89 WT and Ref with codon optimization in the capsid region. After *in vitro* transcription, the four genomic RNAs, including EV-A89 WT, WT/opti, Ref, and Ref/opti, were introduced into RD cell. After serial passaging, we found that the passage 2 (P2) viruses of EV-A89 WT and WT/opti showed obvious CPE, whereas those of EV-A89 Ref and Ref/opti did not (Fig. 2C). In fact, the plaque assay indicated that all 10 passages (P2–P10) of EV-A89 Ref and Ref/opti produced no plaques. Meanwhile, the P2 and P4 viruses of EV-A89 WT and WT/opti induced evident plaques (Fig. 2D). Further, we harvested the P4 viruses of EV-A89 WT and WT/opti and identified the sequences of the viral genome. The sequencing results showed that the capsid sequences of the rescued viruses were identical with those of infectious clones (Fig. 2E). Thus, our results indicated that the four substitutions in the rEV-A89 capsid were indispensable for viral infection.

### The SRIPs of EV-A89 confirmed that the capsid substitutions affected the viral infection and not viral assembly

**Fig 3:**
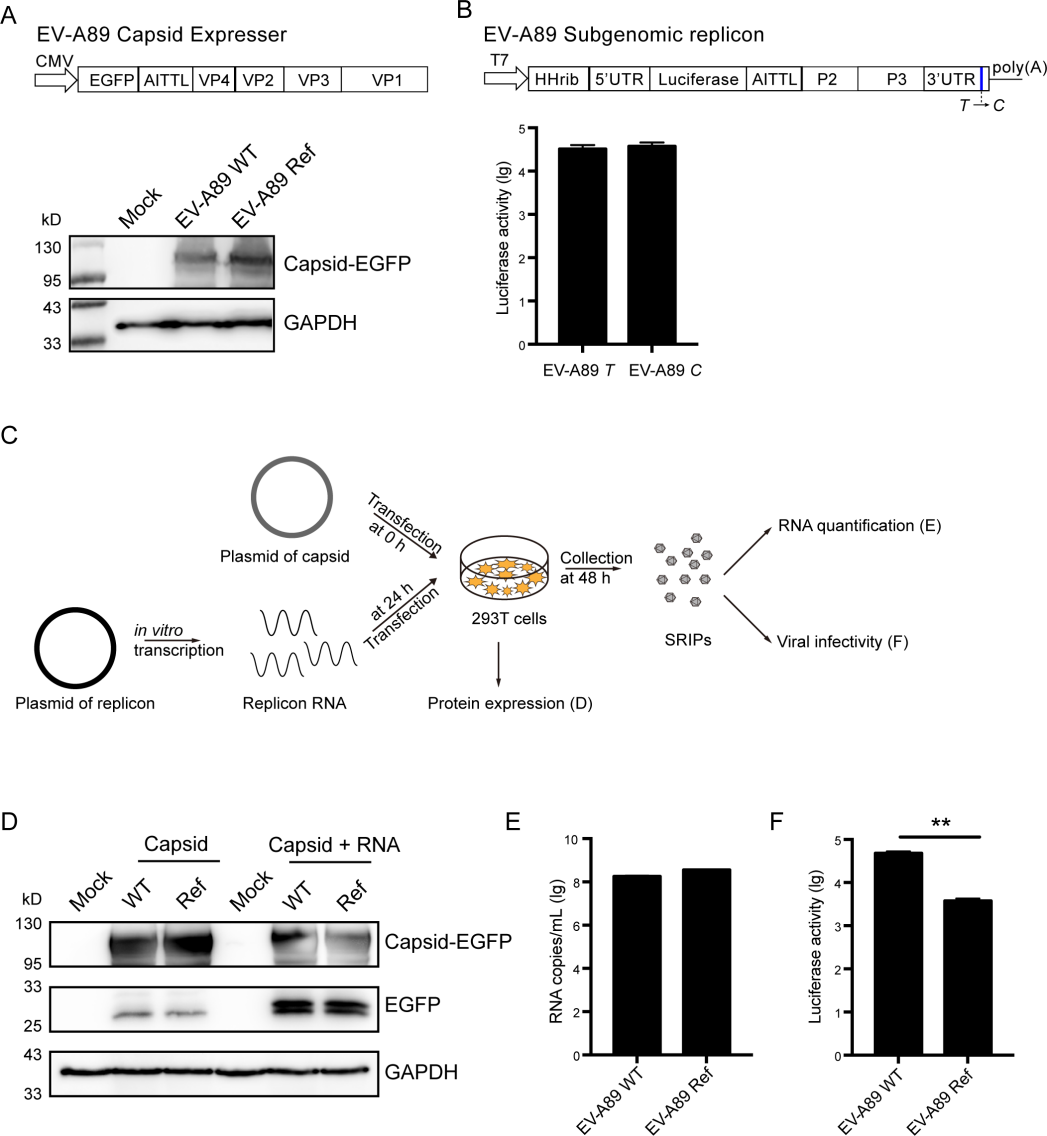
The mutations on EV-A89 capsid increased viral infection. (**A**) Construction of EV-A89 capsid plasmids and Western blot analysis of capsid expression between EV-A89 WT and Ref. The plasmids of EV-A89 WT and Ref capsid fused with EGFP were transfected into 293T cells. At 48 h post-transfection, cells were lysed and probed by anti-green fluorescent protein (GFP) antibody. glyceraldehyde 3-phosphate dehydrogenase (GAPDH) was used as an internal control. (**B**) Construction of EV-A89 subgenomic replicon plasmids and analysis of the replication activity of replicon RNAs. RD cells were transfected with 100-ng replicon RNAs transcribed *in vitro* from EV-A89 replicon plasmids. The activity of the replicon RNA was measured by luciferase activity at 16 h post-transfection. (**C**) The strategy to establish EV-A89 SRIPs. The plasmids of EV-A89 WT or Ref capsids and EV-A71 replicon RNA were subsequently transfected into RD cells. SRIPs were collected at 48 h post-transfection. (**D**) Western blot analysis of the viral protein expression during SRIPs production. After subsequent transfection of capsid plasmids and replicon RNAs, 293T cells were lysed and probed by anti-GFP antibody. GAPDH was used as an internal control. (**E**) RNA genome copies of SRIPs of EV-A89 WT and Ref measured by qRT-PCR. The SRIPs of EV-A89 WT and Ref were treated with RNase for 2 h at 37°C and then proceeded to qRT-PCR. (**F**) Infectivity of SRIPs of EV-A89 WT and Ref. The SRIPs of WT and Ref were applied to RD cells. Luciferase activity was measured at 16 hpi. The significance levels are indicated by **P < 0.01.

To explore the functional implications of these mutations, we employed a subgenomic replicon and single-round infectious particles for EV-A89, bypassing the need for generating viable recombinant viruses. The construction involved a plasmid expressing the EV-A89 capsid protein and an EV-A89 subgenomic replicon plasmid. According to the previous studies (29), we constructed the capsid-expressing plasmid containing all capsid protein genes (VP1–VP4) and an enhanced green fluorescent protein (EGFP) reporter gene under the control of a CMV promoter (Fig. 3A). To explore the possibility of the mutations affecting protein expression, we assessed the expression levels of both EV-A89 WT and Ref capsids in mammalian cells. Our findings observed the comparable expression of capsids fused with EGFP between the two strains, eliminating the influences of mutations on capsid protein expression (Fig. 3A). The subgenomic replicon plasmid consisted of 3′ and 5′-UTRs, a firefly luciferase reporter gene, and all non-structural protein regions, with a T7 promoter sequence inserted before the 5′-UTR (Fig. 3B). It has been previously reported that replicon RNA transcribed from the replicon plasmid using T7 polymerase can efficiently replicate in host cells. As demonstrated in Fig. 3B, the two replicon RNAs of EV-A89 with either *T* or *C* in the 3′-UTR replicated similarly upon transfection into RD cells, indicating the “*T* to *C*” nucleotide mutation did not affect the viral replication.

Prior studies have reported that the subgenomic replicon can assemble into viral particles when provided with viral structural proteins *in-trans*. This approach has been employed to generate SRIPs for several enteroviruses, such as poliovirus (29), EV-A71 (27) and CV-A10 (22). The tropism of SRIPs is determined by the trans-complemented capsids they possess. Thus, SRIPs offer a valuable tool for studying viral tropism and entry mechanisms. We also previously reported successful trans-encapsidation between conventional and unconventional *EV-A* clusters (8). Here, we generated SRIPs of EV-A89 using the EV-A89 capsid in combination with EV-A71 replicon RNA. Subsequently, we established SRIPs for both EV-A89 WT and Ref strains (Fig. 3C). During the production of SRIPs, the capsid-EGFP fused protein of EV-A89 WT and Ref were cleaved successfully, indicating the presence of viral 2A protein (Fig. 3D). To investigate whether the capsid mutations might affect viral assembly and release, we quantified the genome copy numbers of viral RNA of EV-A89 WT and Ref SRIPs through quantitative RT-PCR. The comparable genome copies between EV-A89 WT and Ref showed that viral assembly and release were not affected (Fig. 3E). However, we observed that the infection efficiency of EV-A89 Ref SRIPs was significantly lower compared to that of WT SRIPs (Fig. 3F). In summary, we confirmed that the amino acid substitutions in the EV-A89 WT capsid region increased viral infection by using the SRIPs system.

### The mutations on EV-A89 capsid increased the cell-binding ability

**Fig 4:**
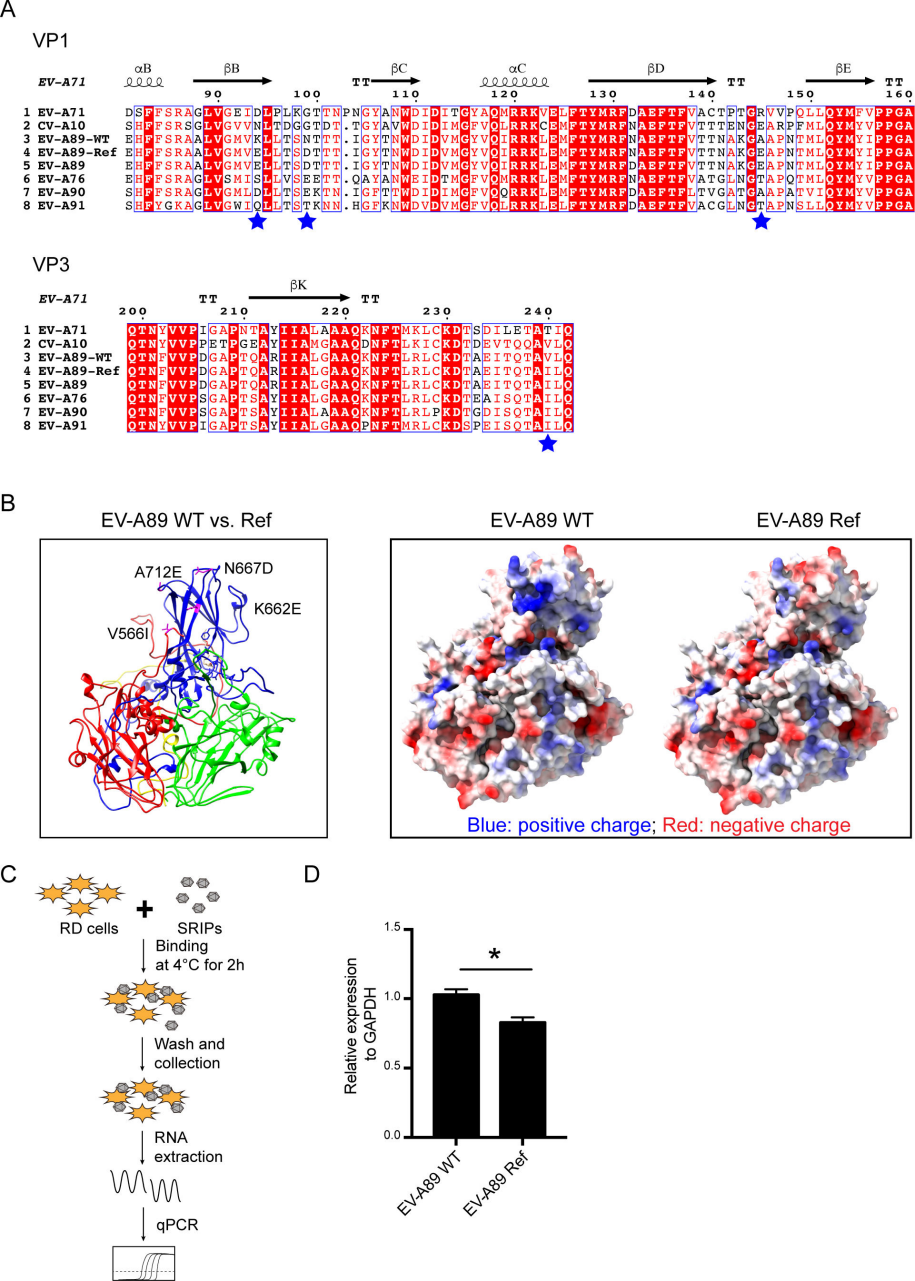
The mutations on EV-A89 capsid increased cell-binding ability. (**A**) Espript 3.0 representation of sequence alignment of VP1 and VP3 of EV-A71 (U22521) with CV-A10 (AY421767), EV-A89 WT, EV-A89 Ref, EV-A89 (AY697459), EV-A76 (AY697458), EV-A90 (AY697460) and EV-A91 (AY697461). Secondary structural elements were labeled. The blue pentacle indicated the amino acid substitutions between EV-A89 WT and Ref. (**B**) The structural homology modeling of EV-A89 protomer (left panel). VP1, blue; VP2, green; VP3, red; VP4, yellow; Mutant sites, magenta. The calculation of residue charges from EV-A89 protomer is shown in right panel. Red, negative charge; Blue, positive charge; White, no charge. (**C**) The carton depicted the binding assay. RD cells were incubated with ~10^8^ genome copies of SRIPs of EV-A89 WT or Ref at 4°C for 2 h. Then cell-bound SRIPs were quantified by RT-qPCR and binding ability of Ref SRIPs was normalized to that of WT SRIPs. (**D**) Binding ability of EV-A89 WT and Ref SRIPs to RD cells. The significance levels are indicated by *P < 0.05.

To gain further insights into the mechanism by which the four mutations facilitated viral infection, we firstly analyzed the location of these four mutations in the secondary structure of the capsid protein. Multiple sequence alignment and comparative analysis of the protein was carried out using ClustalW and visualization was created using ESPript version 3.0 (31). According to the secondary structure of EV-A71 capsid region (32), we found the V566I was located at the C-terminus of VP3 protein, while the K662E/N667D and A712E were at the BC loop and DE loop of VP1 protein, respectively (Fig. 4A). Furthermore, employing structural homology modeling against CV-A6 (PDB code 7QW9), we found that these four substitutions exhibited clustering around the icosahedral 5-fold vertex (Fig. 4B). Intriguingly, our investigation into the charge calculations of the altered capsid residues indicated that the 5-fold vertex in the WT configuration carried a positive charge, while the corresponding region in the Ref configuration was marginally negatively charged (Fig. 4B). Notably, prior studies have underscored the significance of the positively charged fivefold vertex in enteroviruses, as it plays a role in attaching the virus to heparin sulfate glycosaminoglycans on the surface of target cells (33, 34). Thus, these observations strongly suggested a plausible involvement of these four substitutions in the virus’s attachment to host cells. Consequently, we delved into investigating the binding ability of SRIPs of EV-A89 WT and Ref strains to RD cells using a binding assay (Fig. 4C). Indeed, despite adding equal amount of SRIPs, the binding ability of EV-A89 Ref was significantly lower than that of WT (Fig. 4D). These findings suggest that the four substitutions in the EV-A89 capsid influenced viral infection by affecting its binding ability to host cells.

### Mutagenesis analysis unveiled the significance of V566/N667 and K662/A712 in viral infection and cell binding

**Fig 5:**
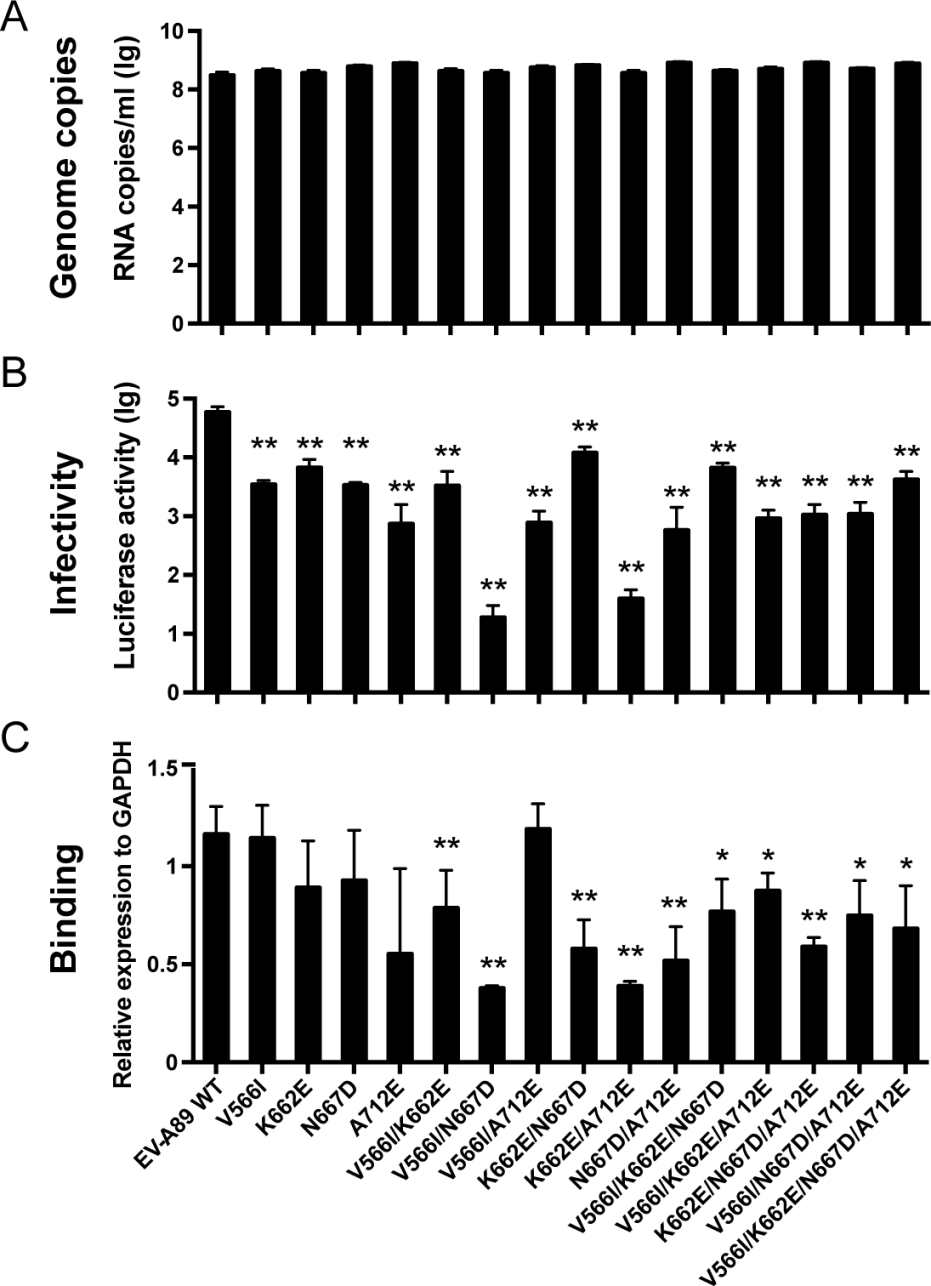
Mutagenesis analysis revealed V566/N667 and K662/A712 were key sites for viral infection and cell binding. (**A**) RNA genome copies of each mutant SRIP were measured by qRT-PCR. (**B**) Mutant viral infectivity measurement. 10^7^ genome copies of each mutant SRIPs were applied to RD cells. Luciferase activity was measured at 16 hpi. (**C**) Mutant SRIPs binding to RD cells. RD cells were incubated with ~10^8^ genome copies of each mutant SRIP at 4°C for 2 h. Then, cell-bound SRIPs were quantified by RT-qPCR, and the binding ability of mutant SRIPs was normalized to that of WT SRIPs. The significance levels are indicated by *P < 0.05, **P < 0.01.

To further identify the pivotal amino acids at these four sites, we conducted mutagenesis by individually or multiply mutating the four amino acids using the EV-A89 WT capsid as a template. A total of 15 mutations were made. Subsequently, SRIPs were produced for each mutant and assessed for viral binding and infectivity. Analysis of the viral RNA genome copies in the mutant SRIPs indicated that the mutations did not impede virus assembly and release (Fig. 5A). However, the SRIPs harboring V566I/N667D and K662E/A712E mutations displayed almost complete loss of viral infectivity and significantly reduced viral binding to RD cells (Fig. 5B and C). Moreover, other mutant SRIPs also exhibited significantly lower viral infectivity compared with EV-A89 WT. Taken together, these results highlight the critical importance of V566/N667 and K662/A712 residues in the capsid for EV-A89 infection and cell binding.

## DISCUSSION

With the global eradication efforts targeting polioviruses, the surveillance for AFP cases has led to the identification of newly discovered non-polio enteroviruses such as EV-A76, EV-B81, EV-B85, and EV-C96, among others. EV-A89 was detected during AFP case surveillance and was identified in China in 2015 (15). Since 2005, EV-A89, along with EV-A76, EV-A90, and EV-A91, has been classified as a distinct phylogenetic clade within the *EV-A* species (7). Notably, our previous studies have revealed that the unconventional *EV-A* clade exhibit very unique virological properties that set them apart from other conventional *EV-A* serotypes (8, 30). Thus, despite the limited range of transmission and population exposure, further in-depth study on the biological and pathogenic properties of *EV-A* serotypes, like EV-A89, is warranted.

Reverse genetics serves as an indispensable tool for investigating various aspects of viral biology. Previously, we successfully established reverse genetic tools for CV-A10, including a Myc-tagged CV-A10 infectious clone (22) and a Nano-Luc reporter CV-A16 (35). In the present study, we have, for the first time, constructed the infectious clone for EV-A89 and successfully rescued the recombinant viruses. The biological characterization of the recombinant EV-A89 was found to be similar to that of the parental virus. Notably, we have attempted but failed to construct the EV-A89 infectious clone by synthesizing the whole genome based on the sequences obtained from NCBI. Here, the alignment of the genomic sequences of the wild type and reference EV-A89 revealed four substitutions in the capsid region, and further investigations confirmed the crucial role of these mutations. As with RNA viruses, the *EV-A* genomes exhibit a high mutation rate, and EVs undergo adaptive mutations during replication *in vitro*. For instance, Arita et al. isolated a mouse-adapted EV-A71 strain with the change of the G (glycine) at position 145 of VP1 to E (glutamic acid). This mutation attenuated the viral infectivity on RD cells but improved viral spread in NOD/SCID mice (36). Furthermore, EV-A71 with VP2-K149I or VP2-K149M adaptive mutations enabled its infection and replication on mouse and hamster cells, overcoming the virus’s inherent non-infectiousness on these cell lines (37, 38). Similar adaptive mutations have been observed in the cytoadaptive culture of CV-A6 and CV-A10, which render the viruses more infectious to certain cell types (39, 40). In the case of rEV-A89 of this study, whether these mutations are adaptive mutations acquired through continuous replication and passage on cells need to be ascertained through continuous culture and assessment from the original isolate.

The reverse genetics system represents a powerful tool for studying the functional implications of mutations. In addition to the infectious clone, we have made plasmids for the EV-A89 capsid protein and a subgenomic replicon. The subgenomic replicon RNA can replicate in permissive cells, serving as a useful indicator of replication-competent cells and facilitating anti-viral drug screening. Subsequent transfection of the capsid expression plasmid and replicon RNA allows the generation of SRIPs, which are valuable for studying viral entry and screening entry inhibitors. Leveraging these tools, we have confirmed the crucial role of these four substitutions in recombinant virus competence. For example, we have identified specific sites that are critical for cell binding and infection, particularly the combination of V566I/N667D and K662E/A712E, while these mutants did not affect the expression and assembly of viral particles. Thus, SRIPs will be a valuable and convenient tool to investigate the role of key residues on *Enterovirus* capsid.

Regarding the underlying mechanism by which these four capsid residues contribute to the enhancement of viral infectivity, our exploration led us to uncover their pivotal clustering around the icosahedral fivefold vertex through structural homology modeling. This clustering strongly suggests their participation in the attachment process to host cells. Intriguingly, three out of the four residues (K662E/N667D and A712E) within the EV-A89 VP1 protein change into negatively charged resides in the Ref counterpart, leading to a disruption of the net positivity present in the fivefold vertex. Numerous previous reports have consistently emphasized the critical role of a positively charged fivefold vertex in the context of virus binding to heparin sulfate glycosaminoglycans, which serve as attachment receptors for a variety of enteroviruses (33, 34, 41, 42). Although we have not conducted direct assays to compare the binding interactions of the WT and Ref capsids with heparin sulfate, we did confirm that the WT SRIPs have superior cell-binding capabilities than their Ref counterparts. Thus, on the basis of these findings, we propose that the four identified substitutions play consequential roles in cell attachment, likely due to their involvement in heparin sulfate binding. This involvement, in turn, promotes viral infection during continuous passaging on cells.

In conclusion, we have successfully constructed the infectious cDNA clone and SRIPs for EV-A89, allowing us to investigate the biological properties of recombinant viruses. Moreover, we have identified key amino acid residues that facilitate EV-A89 infection and elucidate their roles in enhancing viral binding to host cells. The establishment of the reverse genetics system will greatly facilitate future study on the life cycle of EV-A89 and contribute to the development of prophylactic vaccines and anti-viral drugs.

## MATERIALS AND METHODS

### Cells and virus

Human embryonic kidney 293T (HEK293T) cells and human RD cells were maintained in Dulbecco’s modified Eagle’s medium (DMEM, Corning, NY, USA) supplemented with 10% fetal bovine serum (FBS; Gibco, Waltham, MA, USA), 100-U/mL penicillin, and 100-µg/mL streptomycin. The 293 T cells and RD cells were from the Cell Bank of the Chinese Academy of Sciences (Shanghai, China). EV-A89 wild-type strains were propagated in RD cells. Viral titer was determined in RD cells by plaque assay.

### Reagent and antibodies

Lipofectamine 3000 transfection reagent was obtained from Thermo Fisher Scientific (Waltham, MA, USA). Fugene HD transfection reagent was from Promega (Madison, WI, USA). Mouse anti-GFP antibody was from Santa Cruz Biotechnology (Dallas, Texas, USA). Mouse anti-dsRNA antibody was from SCICONS (J2 mAb, Budapest, Hungary). Alexa Fluor 594-conjugated AffiniPure Goat Anti-Mouse IgG (H + L) was purchased from ZSGB-BIO (Beijing, China). Fluoroshield Mounting Medium with DAPI was from Abcam (Cambridge, UK). Luciferase assay system was from Promega. Micrococcal nuclease was from New England BioLabs (Ipswich, MA, USA).

### Cloning strategy

The EV-A89 genome RNA was extracted from a viral supernatant by QIAamp Viral RNA kit (Qiagen, Germany) and then applied to cDNA synthesis following the instruction of GoScript Reverse Transcription System (Promega). The two segments (5′-UTR-P1 and P2-P3-3′-UTR) covering the EV-A89 genome were amplified from the cDNA and seamlessly cloned into the PL451 vector by recombination according to ClonExpress II One step cloning kit (Vazyme Biotech, Nanjing, China). The EV-A89 capsid segment was seamlessly cloned into pcDNA6.0-EGFP to form the EV-A89 capsid expresser. The construct of the EV-A89 replicon was derived from the plasmid of the EV-A71 replicon. Firstly, the original EV-A71 5′-UTR was replaced by EV-A89 5′-UTR. Secondly, the non-structural gene segments of EV-A71 (from 2A to 3′-UTR) were replaced by those of EV-A89. Both plasmids of the infectious clone and replicon had an HHrib (Hammerhead ribozyme) ahead of the 5′-UTR, which would increase the efficiency of replication (43). All the primers used in the cloning strategy are listed in Table S1.

### *In vitro* transcription

The plasmids of EV-A89 infectious clone and replicon were linearized by *Sal*I (NEB, Ipswich, MA, USA) and then purified by PCR Purification Kit (Qiagen). The linearized plasmids were transcribed by T7 polymerase following the manual of T7 High Yield RNA Transcription Kit (Vazyme Biotech). The RNA quality was confirmed by agarose gel electrophoresis.

### RNA transfection

EV-A89 replicon RNA was introduced into RD cells using Lipofectamine 3000 transfection reagent according to the manufacturer’s instruction. RD cells were seeded at 5 × 10^4^ cells/well in 96-well plates. On the next day, the mixture containing 100 ng of *in vitro* transcribed RNA and transfection reagents was added into RD monolayers (90% confluence) and incubated for 16 h at 37°C.

### Plaque assay

The plaque assay was performed using six-well plates containing RD cell monolayers. Ten-fold series of viral dilutions were added, and the plate was shaken every 15 min within 1 h. Then, the inocula were removed, and 2 mL of DMEM containing 2% FBS and 1.2% Avicel (Promega) was added to each well before incubation at 37°C. After being cultured for 3 days, the cells were stained with 0.1% crystal violet (Sigma, St. Louis, USA) containing 10% formaldehyde. Plaques were counted to measure the viral titer.

### Immunofluorescence assay

RD cells were seeded in a 12-well plate (3 × 10^5^ cells/well) with coverslips. On the next day, RD cells were incubated with EV-A89 or rEV-A89 at an MOI of 1 for 7 h. Then, RD cells were washed with cold PBS. After fixation with 4% paraformaldehyde for 15 min, the cells were permeabilized with 0.05% Triton X-100 (Sigma) in 2% FBS/PBS for 10 min. After washes with wash buffer (0.01% Triton X-100 plus 2% FBS in PBS), the cells were blocked for 30 min. Then, the cells were stained with mouse anti-dsRNA antibody (1: 250 dilution, J2 clone) for 1 h at room temperature. Three washes with wash buffer were followed by 30-min incubation with the Alexa Fluor 594-conjugated secondary antibodies. After washes, coverslips were stained by mounting medium with DAPI. Immunofluorescent imaging was taken on TissueFax 200 flow-type tissue cytometer (TissueGnostics GmbH, Vienna, Austria).

### Single-round infectious particles

SRIPs of EV-A89 were generated by sequential transfection of HEK293T cells with the EV-A89 capsid expresser and the EV-A71 replicon RNA. The EV-A89 capsid expresser was firstly transfected by Fugene HD transfection reagent into HEK293T cells at 60%–80% confluence. Twenty-four hours later, EV-A71 replicon RNA was transfected by Lipofectamine 3000 subsequently. The SRIPs were harvested at 24 h post-RNA transfection with two rounds of freeze-thaw cycle.

### Luciferase measurement

For the SRIP infection, ~10^7^ copies of each SRIP mixed with culture medium were applied to RD cells in 96-well plates. The infectivity of SRIPs was scored by luciferase activity using the luciferase assay kit after 16-h infection at 37°C. Briefly, the inocula were removed after 16 h incubation, and then the cells were washed twice by PBS and lysed directly on the plates by the addition of 20 µL of cell culture lysis reagent per well. Then 40 µL of substrate per well was added to the cell lysate, and luciferase activity was measured by Promega GloMax immediately.

### Quantification of SRIPs by measuring viral genome RNA

EV-A89 WT and mutant SRIPs were produced on 293T cells and frozen at −80°C. For viral RNA quantification, 140 µL of each SRIP was used for RNA extraction by using the Viral RNA isolation kit according to the manufacturer’s instruction. The qPCR was performed by using the RNA templated and HiScript II One Step qRT-PCR SYBR Green Master Mix (Q221-01, Vazyme Biotech) with the primers fLuc-F (CAAATACGATTTATCTAATTTACACGA) and fLuc-R (CCGGTATCCAGATCCACAAC) located in the luciferase gene.

### Homology modeling

Homology modeling was created with Swiss Model, and the molecular structures were further analyzed and visualized in the UCSF Chimera version 1.17.3 software (44). The calculation and visualization of charges of the amino acids from EV-A89 WT and Ref capsids were performed by using UCSF ChimetaX (45, 46).

### Binding assay

RD cells were seeded to a 12-well plate (3 × 10^5^ cells/well). On the next day, the cells were incubated with ~10^8^ copies of each SRIP for 2 h at 4°C. Then cells were washed with cold PBS three times to remove unbound SRIPs. Bound SRIPs were quantified by RT-qPCR using the primers fLuc-F and fLuc-R. GAPDH expression was used as an internal control (GAPDH-F: CTGGGCTACACTGAGCACC, GAPDH-R: AAGTGGTCGTTGAGGGCAATG). The expression level of luciferase of each mutant SRIP was normalized to that of WT SRIPs.

### Statistical analysis

All data were expressed as the arithmetic means ± SD of three independent experiments. Significance of differences was evaluated using unpaired Student’s *t*-test. Statistical analysis was performed with GraphPad Prism version 7.0 (La Jolla, CA, USA). The *P* value less than 0.05 was considered to be statistically significant.

## SUPPLEMENTAL MATERIAL

Supplemental figures and tables spectrum.03332-23-s0001.docx Sequence alignment, codon optimization and primers.

ASM does not own the copyrights to Supplemental Material that may be linked to, or accessed through, an article. The authors have granted ASM a non-exclusive, world-wide license to publish the Supplemental Material files. Please contact the corresponding author directly for reuse.
