# Supplementary material for: Construction of an infectious clone for enterovirus A89 and mutagenesis analysis of viral infection and cell binding
Source: Microbiol Spectr. 2024 Mar 5;12(4):e03332-23. doi: 10.1128/spectrum.03332-23 (PMC10986554; doi:10.1128/spectrum.03332-23)
Supplement: Supplemental figures and tables — Sequence alignment, codon optimization and primers. [file spectrum.03332-23-s0001.docx]

Supplemental material


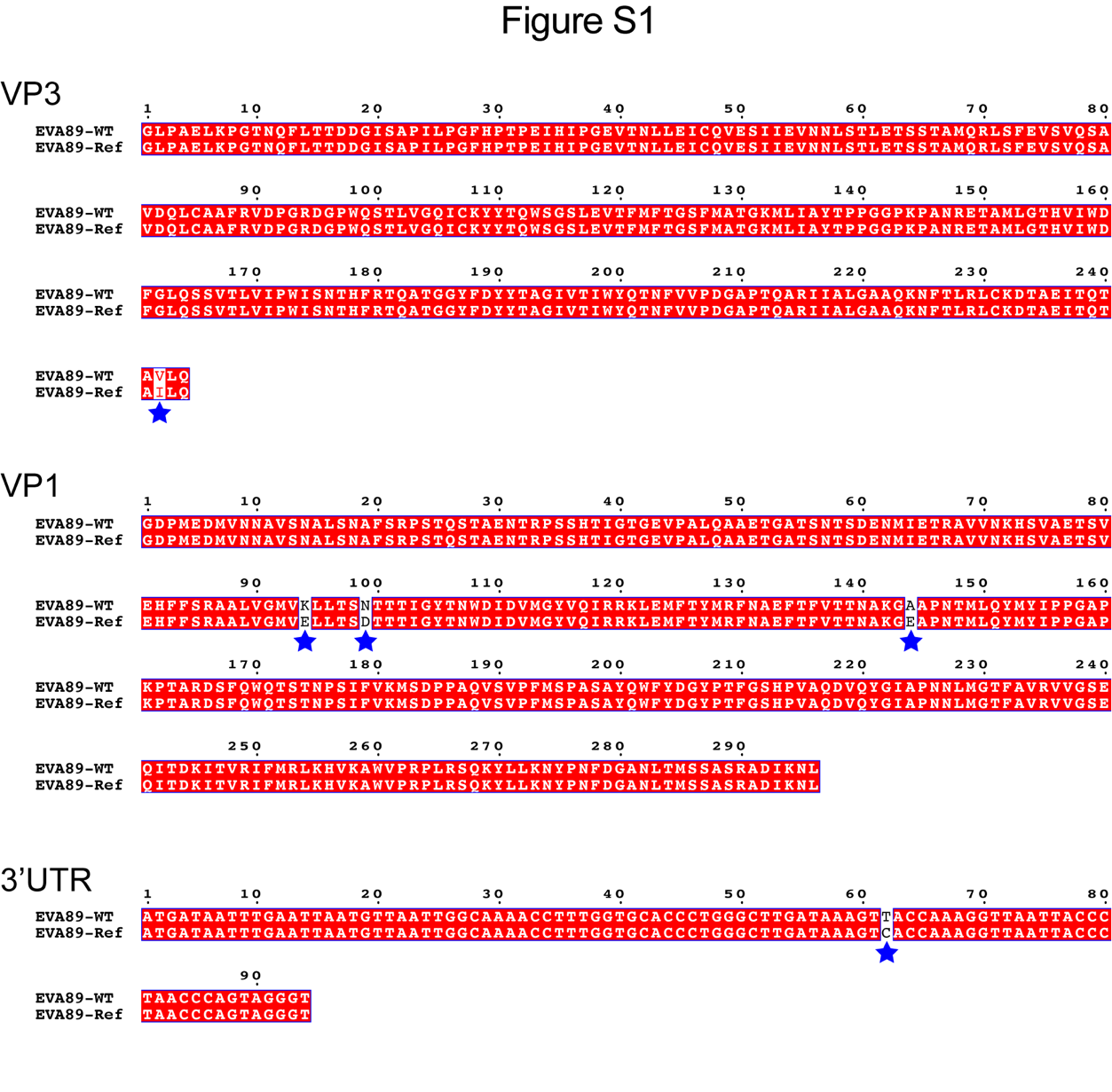


**Figure S1.** **Espript 3.0 representation of sequence alignment of VP1, VP3 and 3’UTR of EVA89 WT and Ref.** The blue pentacle indicated the mutant amino acids and nucleotide.


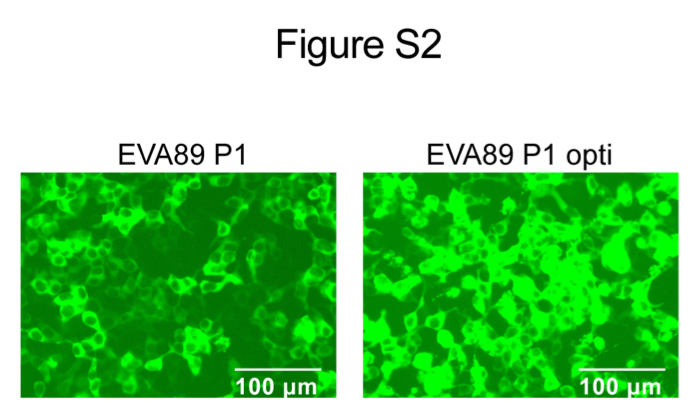


**Figure S2. Codon optimization increased the expression of P1.** The 293T cells were transfected with capsid plasmids and EGFP signals were captured using fluorescence.

**Supplementary Table 1**

The primers for EV-A89 infectious clone, EV-A89 capsid expresser and EV-A89 subgenomic replicon plasmid

| Primers | Sequences |
| --- | --- |
| F1-F | CCGGTATCCCGGGTTCTTAAAACAGCCTGTGGGTTGTACCC |
| F1-R | TTGTTGTCCAAATTTACCAAGATTTTTGATGTCAGCTCGACTGG |
| F2-F | GGTAAATTTGGACAACAATCAGGAGCG |
| F2-R | AACATGAGAATTGTCGACTTTTTTTTTTTTTTTTTTTTTTTTTACCCTACTGGGTTAGGGTAATTAACC |
| PL451-F | GTCGACAATTCTCATGTTTGACAGCT |
| PL451-R | GAACCCGGGATACCGGGTT |
| P1-F | GGCCATTACTACCCTTGGCGCTCAGGTGAGCAC |
| P1-R | GTGATGATGACCGGTTTACAGGTTTTTGATGTCGGCCCG |
| pcDNA6.0-F | TAAACCGGTCATCATCACCATCACC |
| pcDNA6.0-R | AAGGGTAGTAATGGCCTTGTACAGCTCGTCCATGCC |
| 5’UTR-F | CCGGTATCCCGGGTTCTTAAAACAGCCTGTGGGTTGTACCC |
| 5’UTR-R | TTTCTCTTTCTATTGTTTAACAACAAGATGTAAAGGAG |
| P2-3’UTR-F | GGTAAATTTGGACAACAATCAGGAGCG |
| P2-3’UTR-F | AACATGAGAATTGTCGACTTTTTTTTTTTTTTTTTTTTTTTTTACCCTACTGGGTTAGGGTAATTAACC |
| pSVA-F1 | TTAAACAATAGAAAGAGAAAATGGAAGACGCCAAAAACATAAAGAAAG |
| pSVA-R1 | GAACCCGGGATACCGGGTT |
| pSVA-F2 | GTCGACAATTCTCATGTTTGACAGCT |
| pSVA-R2 | TTGTTGTCCAAATTTACCAAGGGTAGTAATGGCCAATTTGGAC |
